# Coexistence of the suprascapular notch and the suprascapular foramen—a rare anatomical variation and a new hypothesis on its formation based on anatomical and radiological studies

**DOI:** 10.1007/s12565-012-0165-7

**Published:** 2012-12-04

**Authors:** Michał Polguj, Kazimierz Jędrzejewski, Agata Majos, Mirosław Topol

**Affiliations:** 1 Department of Angiology, Medical University of Łódź, Narutowicza 60, 90-136 Łódź, Poland; 2 Department of Normal and Clinical Anatomy, Medical University of Łódź, Łódź, Poland; 3 Radiology Department, Medical University of Łódź, Kopcińskiego 22, 90-153 Łódź, Poland

**Keywords:** Suprascapular foramen, Suprascapular notch, Anterior coracoscapular ligament, Suprascapular nerve entrapment

## Abstract

The suprascapular notch is the most common site of suprascapular nerve entrapment, which can manifest in disability and pain of the upper limb. Here, we present three cases of a very rare anatomical variation in the suprascapular region: the coexistence of the suprascapular notch and the suprascapular foramen. The variation was found during radiological and anatomical investigations. The suprascapular foramen was situated inferior to the suprascapular notch. A bony bridge lay between them, likely created by an ossified anterior coracoscapular ligament (ACSL). This anatomical variation probably increased the risk of suprascapular nerve entrapment by nerve irritation of the bony margins during passsage through the foramen and by a lack of the elasticity that the ACSL normally demonstrates. Also, a bony bridge passing through the middle part of the suprascapular notch reduces the space available for nerve passage (bony bridge decreases the space by about 36.5–38.6 %). One patient who underwent the radiological study had typical symptoms of suprascapular nerve entrapment. Based on his medical history and the presence of this rare variation of the suprascapular notch at the suprascapular region we suspect this neuropathy.

## Introduction

The suprascapular nerve (SN) passes through the suprascapular notch (SSN) inferior to the superior transverse scapular ligament (STSL). The suprascapular region is characterised by a plurality of anatomical variations, including variations in the alignment of the nerve and vessels as well as the morphology of the bony incisura and ligaments attached to its margins. The risk of a suprascapular neuropathy can be increased by anatomical anomalies such as an ossified or bifid STSL (Duparc et al. 2010; Polguj et al. 2011), a narrow “V” shape of the suprascapular notch (Rengachary et al. 1979), a hypertrophied subscapular muscle (Duparc et al. 2010) or the presence of an anterior coracoscapular ligament (ACSL) (Avery et al. 2002; Bayramoğlu et al. 2003). The main cause of this pathological condition is a narrowing of the osteofibrous tunnel for passage of the suprascapular nerve, resulting in a mechanical irritation of the nerve during shoulder movements. Such mechanical irritation is responsible for 0.4–2 % of cases of shoulder girdle pain (Gosk et al. 2007; Post and Mayer 1987). An early decompression of the nerve by resection of the STSL has proven to be a safe and effective procedure that relieves pain and restores limb function (Lafosse et al. 2007). Consequently, knowledge of these anatomical variations is of paramount importance when a decision must be made as to which procedure to use—an open, arthroscopic or arthroscopically assisted method (Urgüden et al. 2004).

## Case reports

Three unique anatomical variations at the suprascapular region were observed during radiological and anatomical studies. In all cases, a suprascapular notch coexisted with a suprascapular foramen, with the latter situated below the former, and a bony bridge lay between them. To better present this variation, we made several measurements.

### Collected measurements

**Fig. 1:**
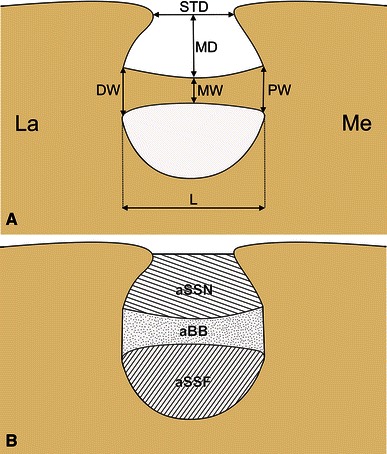
Schematic representation of the arrangements of the structures in the suprascapular region of the scapula with the coexistence of a suprascapular notch and suprascapular foramen. *La* Lateral, *Me* medial. **a** Measurements of the suprascapular notch: *MD* maximal depth, *STD* superior transverse diameter; measurements of the bony bridge: *L* length, *PW* proximal width, *MW* middle width, *DW* distal width. **b** Measurements of the areas in the suprascapular region: *aBB* area of the bony bridge, *aSSF* area of the suprascapular foramen, *aSSN* area of the suprascapular notch

The following measurements of the suprascapular notch were evaluated (Fig. 1a):

1. The superior transverse diameter (STD), as the distance between the proximal and distal margins of the SSN, along the upper border of the scapula.
2. The maximal depth (MD), as the distance between the middle point of an imaginary line connected the superior corners of the notch to the deepest point of the suprascapular notch.

The following measurements of the bony bridge were collected (Fig. 1a):

1. Length (L), as the distance between proximal and distal ends of the bony bridge.
2. Proximal width (PW), as the distance between the superior and inferior borders of the bony bridge at its proximal end.
3. Middle width (MW), as the distance between the superior and inferior borders of the bony bridge half way along its length.
4. Distal width (DW), as the distance between the superior and inferior borders of the bony bridge at its distal end.

The following values were measured (Fig. 1b):

1. Area of the suprascapular foramen (aSSF)—the area limited by the osseus border of the suprascapular foramen.
2. Area of the suprascapular notch (aSSN)—the area between the superior border of the bony bridge and the superior transverse diameter (STD) of the SSN.
3. Area of the bony bridge (aBB)—the area limited by the boundaries of the bony bridge.

The following parameters were calculated using these measurements:

1. Real area for passage (raP)-the reconstructed, true area of the possible passage of the suprascapular nerve (without the area of the bony bridge)
  $$ {\text{raP}} = {\text{aSSF}} + {\text{aSSN}} $$
2. Potential area for passage (paP)—the reconstructed, theoretical area for the possible passage of the suprascapular nerve
  $$ {\text{paP}} = {\text{aSSF}} + {\text{aSSN + aBB}} $$
3. Index reduction of passage (IrP)—a determination of the influence of the bony bridge on decreasing the potential area available for the passage the suprascapular nerve (in %)
  $$ {\text{IrP}} = {\text{raP}}/{\text{paP}} \times 100\,\% $$

### Radiological study

We retrospectively analyzed computed tomography (CT) scans of 308 randomised patients obtained using a standard CT chest protocol. Multidetector computed tomography (MDCT) imaging was performed with a 32-row MDCT scanner (Toshiba Aquilion 32; Toshiba Medical Systems, Otawara-shi, Tochigi, Japan). Specimens with metastases to the bone were excluded from the study. The research project and procedures were approved by the Bioethics Commission of the Medical University of Lodz (protocol no. RNN/12/10/KE). All scapulae were analysed with post-processing tools: multiplanar reconstructions (MPR) and maximum intensity projection (MIP) images on the coronal and sagittal planes. Coronal curved MIP images were obtained and three-dimensional (3D) volume rendering (VR) data were acquired. The measurements of the suprascapular region were performed on a 3D reconstruction of the scapula using Vitrea 2 system software (Vital Images, Plymouth, MA).

**Fig. 2:**
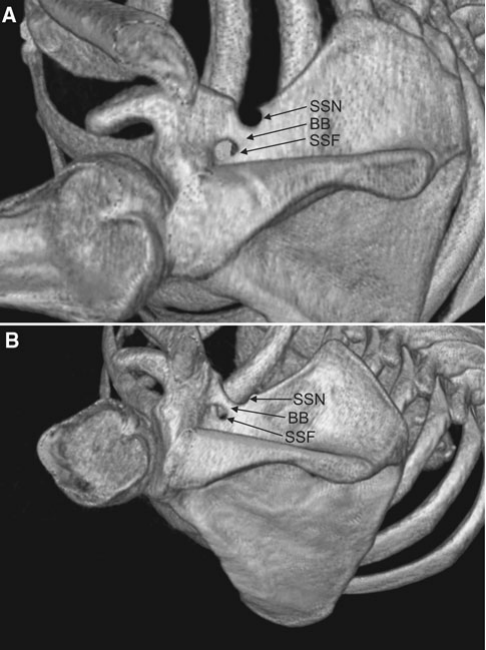
Three-dimensional volume rendering (VR) multidetector computed tomography (MDCT) at suprascapular region of the scapula: *BB* Bony bridge, *SSF* suprascapular foramen, *SSN* suprascapular notch. **a** Case 1, **b** case 2

A unique anatomical variation at the suprascapular region was identified in two of the 616 randomized scapulae analysed by CT (0.33 %) (Fig. 2).

#### Case 1

A 41-year-old Caucasian male was admitted to the surgical department of our hospital for abdominal and chest pain associated with vomiting. A physical examination revealed palpation pain in the left epigastrium. Stomach ulcer was diagnosed by gastrofiberoscopy, and appropriate treatment was initiated. Physical examination also revealed weakness of abduction and external rotation of the left upper extremity. The patient reported suffering from deep and diffused, poorly localized pain of the left shoulder for 5 months, and further examination revealed atrophy of the left supraspinatus muscle. However, he denied even a slight trauma of this region. The right scapula had no bony bridges at the suprascapular region. The patient denied pain or weakness of the right shoulder. The right infraspinatus muscle was normal. Suprascapular neuropathy of the left shoulder was suspected based on the clinical findings.

Further examination by our scientific team identified the coexistence of a suprascapular notch and a suprascapular foramen in the left scapula. However, additional information was not available on this patient because he did not consent to an electromyographic examination to confirm the suprascapular neuropathy.

In this case of a coexisting suprascapular notch and foramen, a 11.3-mm bony bridge was present between the two structures. The width of the bridge in the proximal, middle and distal aspects was 7.2, 5.8 and 8.8 mm, respectively. The maximum depth and superior transverse diameter of the suprascapular notch was 7.1 and 6.8 mm, respectively. The areas of the different parts of the suprascapular notch were: aSSF, 43.4 mm^2^; aSSN, 40.5 mm^2^; aBB, 50.1 mm^2^. The calculated areas of the suprascapular nerve passage were: raP, 83.9 mm^2^; paP, 134.0 mm^2^, respectively (Table 1). The IrP was 37.4 %.

**Table 1:** Measurements of the structures at the suprascapular region in the scapula with coexisting suprascapular notch and suprascapular foramen

Structure	Measurements	Radiological study		Anatomical study
		Case 1	Case 2	
Bony bridge (BB)	Length (L) (mm)	11.3	9.3	12.1
	Width (mm)			
	Proximal (PW)	7.2	6.7	6.4
	Middle (MW)	5.8	4.7	4.6
	Distal (DW)	8.8	7.1	7.9
	Area (mm^2^) (aBB)	50.1	61.6	45.1
Suprascapular foramen (aSSF)	Area (mm^2^)	43.4	39.6	27.3
Suprascapular notch (SSN)	Maximal depth (MD) (mm)	7.1	5.4	4.9
	Superior transverse diameter (STD) (mm)	6.8	12.8	9.5
	Area (mm^2^) (aSSN)	40.5	58.4	51.1
Real area for passage (raP)	Area (mm^2^)	83.9	98.0	78.4
Potential area for passage (paP)	Area (mm^2^)	134.0	159.6	123.5

*aBB* Area of the bony bridge, *aSSN* area of the suprascapular notch

#### Case 2

The coexistence of a suprascapular notch and suprascapular foramen was identified in the left scapula of a 47-year-old Caucasian woman who had been hospitalised because of suspicion of pulmonary embolism (Fig. 2b). No bony bridges at the suprascapular region were observed in the contralateral scapula (right). The patient had no symptoms of suprascapular nerve entrapment syndrome. The bony bridge was 9.3 mm long, and its width was 6.7, 4.7 and 7.1 mm in the proximal, middle and distal aspects, respectively. The maximal depth and superior transverse diameter of the suprascapular notch were 5.4 and 12.8 mm, respectively. The area of the suprascapular foramen was 39.6 mm^2^; the measured areas were: aSSF, 39.6 mm^2^; aSSN, 58.4 mm^2^; aBB, 61.6 mm^2^. The calculated areas of the suprascapular nerve passage were: raP-98.0 mm^2^ and paP-159.6 mm^2^ (Table 1). The IrP was 38.6 %.

### Anatomical study

**Fig. 3:**
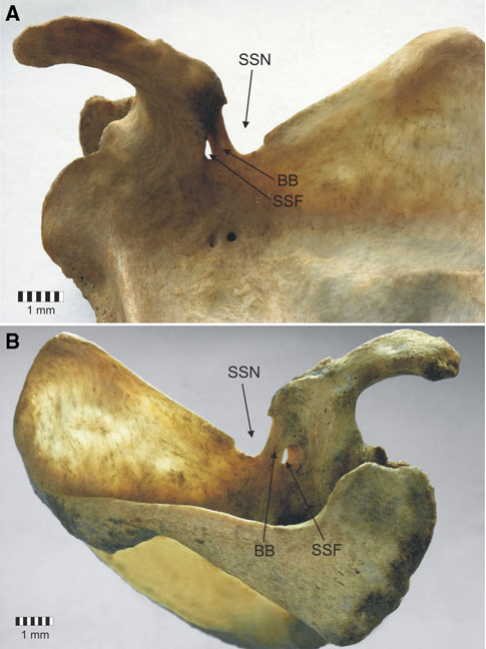
Structures of the suprascapular region: *BB* bony bridge, *SSF* suprascapular foramen, *SSN* suprascapular notch. **a** Antero-superior aspect, **b** postero-superior aspect

A search of the collection of the Department of Anatomy, Medical University of Lodz revealed a right scapula with coexisting SSN and SSF (Fig. 3). All measurements of the structures at the suprascapular region were taken using two complementary but independent methods: (1) a classical osteometry using an electronic digimatic caliper (Mitutoyo Co, Kawasaki, Japan); (2) a new method based on an analysis of digital photographic documentation of the structures at the suprascapular region using MultiScanBase v.14.02 software (Computer Scanning System II, Warsaw, Poland).

The superior transverse diameter and the maximal depth of the suprascapular notch were 9.5 and 4.9 mm, respectively. The bony bridge was 12.1 mm long, and its width in the proximal, middle and distal aspects were 6.4, 4.6 and 7.9 mm, respectively. The measured areas were: aSSF, 27.3 mm^2^; aSSN, 51.1 mm^2^; aBB, 45.1 mm^2^. The calculated areas of the suprascapular nerve passage were: raP, 78.4 mm^2^; paP, 123.5 mm^2^, respectively (Table 1). The IrP was 36.5 %.

## Discussion

The patient from our first radiological study (case 1) suffered from deep and diffuse poorly localized pain of the left shoulder and weakness of abduction and external rotation of the left upper extremity in the glenohumeral joint. Atrophy of the left supraspinatus muscle was also observed. These symptoms are the main clinical manifestations of suprascapular nerve entrapment (Gosk et al. 2007; Post and Mayer 1987; Zehetgruber et al. 2002).

Although suprascapular nerve entrapment is uncommon, it should be considered in the differential diagnosis of patients with shoulder pain and weakness, which includes brachial plexopathy, cervical spine disease, cervical discopathy, glenohumeral joint diseases, tendonitis or bursitis and rotator cuff tear (Gosk et al. 2007; Zehetgruber et al. 2002). The diagnosis of suprascapular nerve entrapment is based on physical examination, medical history and the results of additional investigations (Cummins et al. 2000). The most important of the latter are the results from electrodiagnostic studies, including studies of nerve conduction velocity from Erb’s point to the supra- and infraspinatus muscles and electromyography. The normal latency ranges for the supra- and infraspinatus muscles are 1.7–3.7 and 3.3–4.2 ms, respectively (Kraft 1972; Kullmer et al. 1998).

The patient from our radiological study (case 1) refused further diagnostic evaluation of his shoulder pain. However, based on his clinical presentation and the radiographical findings, we consider that he presented with suprascapular nerve entrapment.

Although many anatomical variants of the suprascapular notch have been reported in the literature, coexistence of the suprascapular notch and the suprascapular foramen has been described only three times (Hrdicka 1942; Natsis et al. 2007; Sinkeet et al. 2010). The first report was that of Hrdlicka who in 1942 found one such case among 2792 dried scapulae (0.036 %). The second report was that of Natsis et al. (2007), who discovered this rare type of anatomical variants in three of 423 scapulae from the German general population (0.7 %). Scientists have classified scapulae with both a notch and a foramen at the site of the SSN as a distinct type (Type V). More recently, there have also been descriptions from Africa; Sinkeet et al. (2010) found one such a case in 138 investigated Kenyan scapulae (0.72 %).

In our study, the frequency of the coexistence of a suprascapular notch and a suprascapular foramen was 0.33 %, which was higher than that reported by Hrdlicka (1942) (0.036 %) and lower than that described by Natsis et al. (2007) in German scapulae (0.7 %) and by Sinkeet et al. (2010) (0.72 %) in the Kenyan population. It would be reasonable to suppose that these frequencies depend on the population, similar to the frequency of complete ossified STSL.

**Fig. 4:**
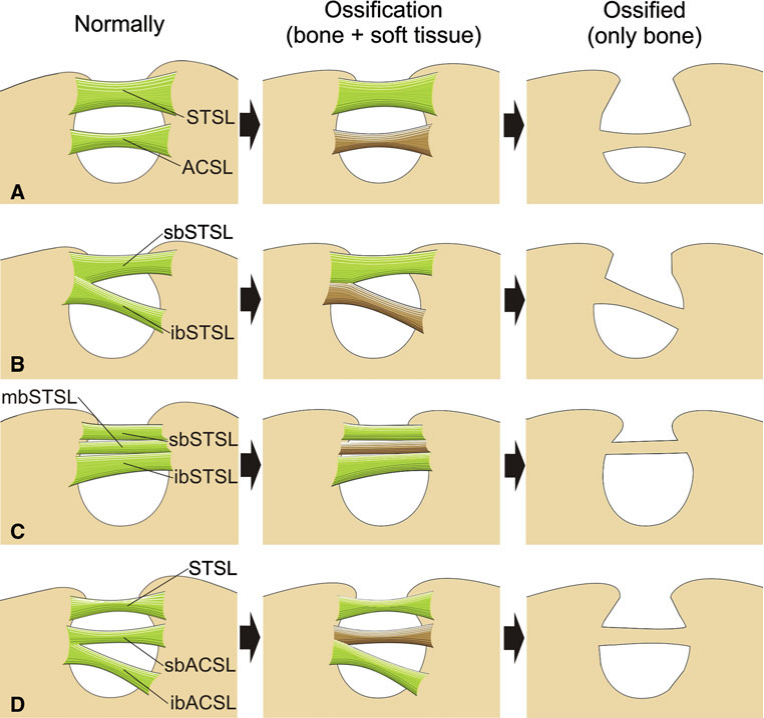
Schematic arrangements demonstrating four hypotheses (**a–d**) explaining the formation of coexisting suprascapular notch and foramen. *STSL* Superior transverse scapular ligament, *sbSTSL* superior band of superior transverse scapular ligament, *mbSTSL* middle band of superior transverse scapular ligament, *ibSTSL* inferior band of superior transverse scapular ligament, *ACSL* anterior coracoscapular ligament, *sbACSL* superior band of anterior coracoscapular ligament, *ibACSL* inferior band of anterior coracoscapular ligament

To our knowledge, no hypotheses have been published to explain the coexistence of a suprascapular notch and foramen. Here, we present four hypotheses on the formation of this phenomenon which are based on the latest anatomical findings (Avery et al. 2002; Bayramoğlu et al^.^ 2003) (Fig. 4).

In our opinion, the first hypothesis (Fig. 4a) is the most probable as it assumes that the ossification of the single bundle ACSL would create the bony bridge above the suprascapular foramen. The osseous transformation of the STSL would not occur, and so it would be absent on a dry scapula, and a suprascapular notch would be formed. The ACSL was first described by Avery et al. (2002) as a fibrous band extending along the anterior aspect of the SSN, just below the STSL. Its proximal and distal attachments insert separately onto the borders of the SSN and run parallel or obliquely to the STSL. The topography of the bony bridge observed in our anatomical and radiological study was similar to that observed by Avery et al. (2002) on the anterior coracoscapular ligament. Therefore, the first hypothesis is the most probable. In our opinion, the ossification of the ACSL can increase the risk of suprascapular neuropathy because of the higher potentiality for nerve irritation by the bony margins of the foramen and the lack of elasticity that the ACSL normally demonstrates. The etiopathology of the suprascapular nerve entrapment proposed by Rengachary et al. (1979), called the “sling effect”, assumes that during motions of the arm, the nerve makes minimal transitional movements. Therefore, an angulated nerve can be pressed against the sharp bony margin when travelling through the suprascapular foramen during the action of the upper limb. The repeated kinking irritates the nerve and induces microtrauma that might result in this neuropathy. Although in the case presented here it is impossible to determine the course of the SN, the area of the suprascapular region was highly reduced in comparison to the potential area of the normal notch without a bony bridge. The frequency of the ACSL has been found to be 18.8 % (Bayramoglu et al. 2003—Turkish population), 28 % (Piyawinijwong and Tantipoon 2012—Thai population) or 60 % (Avery et al. 2002—American population). All scientists confirm its presence as an additional etiological factor of the condition.

The second potential mechanism (Fig. 4b) described in this article which explains the variation is calcification of the inferior part of a bifid STSL. In such a case, the ligament has two bands (superior and inferior) that are separately fixed to the one border of the suprascapular notch. Both parts of this bifid STSL travel independently one below the other, but they have a common opposing attachment. An ossified inferior band of the bifid STSL might create a bony bridge (Fig. 4b). Despite the fact that frequency of the bifid STSL varies from 3.3 to 15.6 % (Bayramoglu et al. 2003; Duparc et al. 2010), partial ossification (not whole) of the ligament seems unlikely.

The third hypothesis (Fig. 4c) explaining the coexistence of a suprascapular notch and foramen depends on incomplete ossification of a trifid STSL. This type of superior transverse scapular ligament has three parts: the superior, middle and inferior band. When only the middle band ossifies, the bony bridge is formed, and the suprascapular notch and foramen are present on dry scapulae (Fig. 4c). A survey of recent literature revealed only two cases of trifid STSL (Polguj et al. 2012; Ticker et al. 1998). In the study of Ticker et al. (1998), the ligament had the middle band partially ossified, possibly confirming this hypothesis. However, Polguj et al. (2012) did not find any signs of ossification in the ligament in their study.

The fourth potential mechanism explaining the coexistence of a suprascapular notch and foramen is the calcification of the superior band of a bifid ACSL (Fig. 4d). However, this explanation seems rather unlikely because, to our knowledge, no case of partially or completely ossified bifid ACSL has ever been reported.

The SSF and the SSN are the most probable passages for the SN. Data obtained from our photographic documentation analysis indicate that the areas of both spaces were considerably decreased by the presence of the bony bridge in comparison to the reconstructed potential area of the suprascapular nerve passage. The space available for the passage was decreased by about 37.4–38.6 (radiological study) and 36.5 % (anatomical study). In our opinion, the coexistence of the suprascapular notch and suprascapular foramen narrows the space for passage of the nerve and, therefore, might also increase the risk of suprascapular neuropathy.
